# Inhibition of melanogenesis by *Aster yomena* callus pellet extract in melanoma cells and patients with skin pigmentation

**DOI:** 10.7150/ijms.62530

**Published:** 2021-07-23

**Authors:** Jae-In Lee, Jeong Hun Seo, Eun-Sil Ko, Sang-Min Cho, Jea-Ran Kang, Jong-Hoon Jeong, Yu Jeong Jeong, Cha Young Kim, Jeong-Dan Cha, Woo Sik Kim, Young-Bae Ryu

**Affiliations:** 1 Functional Biomaterial Research Center, Korea Research Institute of Bioscience and Biotechnology, Jeongeup-si, Jeollabuk-do 56212, Republic of Korea; 2 Department of Bio-material and product development and R&D center, General Bio, Namwon-si, Jeollabuk-do 55793, Republic of Korea

**Keywords:** *Aster yomena*, callus, extract, metabolite, melanogenesis, skin pigmentation

## Abstract

Plant tissue culture holds immense potential for the production of secondary metabolites with various physiological functions. We recently established a plant tissue culture system capable of producing secondary metabolites from *Aster yomena*. This study aimed to uncover the mechanisms underlying the potential therapeutic effects of *Aster yomena* callus pellet extract (AYC-P-E) on photoaging-induced skin pigmentation. Excessive melanogenesis was induced in B16F10 melanoma cells using α-melanocyte stimulating hormone (α-MSH). The effects of AYC-P-E treatment on melanin biosynthesis inducers and melanin synthesis inhibition were assessed. Based on the results, a clinical study was conducted in subjects with skin pigmentation. AYC-P-E inhibited melanogenesis in α-MSH-treated B16F10 cells, accompanied by decreased mRNA and protein expression of melanin biosynthesis inducers, including cyclic AMP response element-binding protein (CREB), tyrosinase, microphthalmia-associated transcription factor (MITF), tyrosinase related protein-1 (TRP-1), and TRP-2. This anti-melanogenic effect was mediated by mitogen-activated protein kinase (MEK)*/*extracellular signal-regulated kinase (ERK) and protein kinase B (AKT) phosphorylation. Treatment of subjects with skin pigmentation with AYC-P-E-containing cream formulations resulted in 3.33%, 7.06%, and 8.68% improvement in the melanin levels at 2, 4, and 8 weeks, respectively. Our findings suggest that AYC-P-E inhibits excessive melanogenesis by activating MEK/ERK and AKT signaling, potentiating its cosmetic applications in hyperpigmentation treatment.

## 1. Introduction

Nature offers incredibly diverse natural substances that are capable of exerting various physiological effects 1. Such natural compounds extracted from plants, animals, or other organisms are widely used as ingredients in personal care products and natural cosmetics 2-4. However, the composition of these substances is greatly affected by environmental factors. For instance, the growth conditions, such as climate or soil parameters, harvest timing, production methods, and storage conditions affect the constitution of natural compounds obtained from plants 5. Moreover, various environmental pollutants (water quality, air, and soil pollution) can result in mutations and organism extinction 6. These phenomena present a significant challenge to the homogeneous extraction of natural substances from a variety of biological species that is essential to the cosmetic industry.

In recent years, several studies have actively utilized natural materials possessing excellent physiological activity in functional cosmetics using plant tissue culture technology 7-10. Plant tissue culture is a sterile culture technique that extracts organs, tissues, and cells from a plant, followed by culturing in medium with nutrients in order to regenerate a plant with complete functionality or isolate groups of single cells from a callus 11. This technique can be used to effectively mass-produce plant cells and organs in a consistent manner through the maintenance, preservation, and periodic subculture of specific cell lines 11-13. In particular, metabolites (including alkaloids, flavonoids, polyphenols, terpenes, triterpenes, cardiac glycosides, paclitaxel and related taxoids) with various physiologically active functions can be produced using this method 14, 15. Therefore, the plant tissue culture technique is actively studied to continuously improve the production of biologically valuable high-quality materials 16-18. Nonetheless, research on the selection and function of plant callus materials that can be used as functional cosmetic ingredients is still lacking.

*Aster yomena*, a perennial herb found in the southern regions of South Korea, is widely used as a functional food ingredient in these regions. Recent studies have shown that extracts isolated from this plant using various methods possess various physiological properties, such as anti-obesity, anti-arthritic, and anti-inflammatory properties 19-21. We have recently succeeded in establishing an *Aster yomena* callus culture system and confirmed that metabolites with various functions can be produced using this system. Importantly, the metabolite composition of *Aster yomena* callus pellet extract (AYC-P-E) was found to alleviate skin photoaging 22. Based on these findings, AYC-P-E is expected to be widely used as a functional cosmetic ingredient capable of preventing skin photoaging. The purpose of this study was to investigate the effects of AYC-P-E on photoaging-induced skin pigmentation. As skin pigmentation is caused by an excessive increase in the melanin content of skin tissues, we first confirmed the anti-whitening effects of AYC-P-E in B16F10 melanoma cells following the induction of excessive melanogenesis using melanin stimulating hormone (α-MSH). Furthermore, based on the *in vitro* findings, we conducted a clinical study of a cosmetic formulation that contains AYC-P-E in 22 healthy subjects with facial hyperpigmentation.

## 2. Materials and methods

### 2.1. Preparation of AYC-P-E

AYC-P-E was prepared according to previously described protocols 22. Briefly, sterilized *Aster yomena* roots were placed into Murashige and Skoog (MS) solid medium supplemented with 1 mg/l 2,4-dichlorophenoxyacetic acid (2,4-D), 3% (w/v) sucrose, and 0.4% (w/v) Gelrit for 2 weeks (at 25°C in the dark) to induce callus formation. The resultant cultured callus was inoculated into a 500 ml flask containing 100 ml of a liquid MS1D medium and cultured at 25°C in the dark on a rotary shaker at 80 rpm. Next, 100 ml cell suspensions for the exponential phase were transferred to a 2 l tank of a sterilized glass bioreactor, and the volume was adjusted to 1.5 l with the liquid MS1D medium. The bioreactor was maintained at 25°C in the dark, and the callus was incubated for 10 days. After 10 days, the pellets were collected following centrifugation at 12,000 rpm for 30 min at 4°C. The collected pellets (5 g) were suspended in 1 l sterile distilled water (DW) and incubated at 80°C for 2 h, and the supernatants were collected following centrifugation at 12,000 rpm for 30 min at 4°C. The collected solutions were dried in a vacuum freeze drier, and the freeze-dried powders were resuspended in PBS and filtered through a 0.22 μm filter. The final concentration of AYC-P-E was adjusted to 30 mg/ml.

### 2.2. Melanoma cell line culture

A cryopreserved B16F10 murine melanoma cell line was obtained from Korea Cell Line Bank (KCLB, Seoul, South Korea). B16F10 cells were grown in the presence of Dulbecco's Modified Eagle's Medium (Gibco BRL, Grand Island, NY, USA) supplemented with 10% fetal bovine serum (Gibco BRL) and 1% penicillin/streptomycin (Gibco BRL) at 37°C in a humidified incubator (5% CO_2_ condition; Thermo Scientific, Waltham, MA, USA).

### 2.3. Cell viability and cytotoxicity

B16F10 cells at passage 5 were used for all the experiments. To investigate the cytotoxic effect of AYC-P-E in melanoma cells, B16F10 cells were seeded in a 6-well plate at a density of 1×10^5^ cells/well for 12 h, and the media were removed. Then, culture medium containing AYC-P-E at various concentrations (15, 30, 60, and 120 μl/ml) was added. After 72 h, cells were harvested and washed twice with phosphate-buffered saline (PBS). The washed cells were stained with Annexin V (diluted 1:50 in Annexin V binding buffer; BD Bioscience, San Diego, CA, USA) and propidium iodide (PI, diluted 1:25 in Annexin V binding buffer; BD Bioscience) for 15 min at room temperature (20°C ± 5) in the dark and then washed with Annexin V binding buffer. Data were acquired using a Life Launch Attune Nxt Flow Cytometer (ThermoFisher Scientific) and analyzed using the FlowJo software (Version 10; Tree Star, Inc., Ashland, OR). Next, to investigate the protective effect of AYC-P-E on melanoma cells against staurosporine (STS)-induced apoptotic damage. B16F10 cells (1×10^5^ cells/well) were seeded in a 6-well plate for 12 h. Then, culture medium containing AYC-P-E (120 μl/ml) and STS (50 nM; Sigma-Aldrich, St. Louis, Mo, USA) was added. After 72 h, cell viability and apoptotic cell death were analyzed using the EZ-Cytox kit (DoGen, Seoul, Korea) and annexin V/PI staining, respectively. Cell viability assay using EZ-Cytox kit was conducted according to the manufacturer's instructions.

### 2.4. Melanin content measurement

B16F10 cells were seeded in a 6-well plate at a density of 5×10^4^ cells/well for 12 h, and the media were removed. Then, culture medium (phenol red-free) containing α-MSH (200 nM; Sigma-Aldrich) was added for 1 h prior to treatment with AYC-P-E for an additional 72 h. To determine the extracellular melanin content, culture supernatants were analyzed at 405 nm using a microplate reader (Molecular Devices Inc., San Jose, CA, USA). To determine the intracellular melanin content, the cultured cells were lysed using RIPA buffer (Pierce, Rockford, IL, USA) and centrifuged at 12,000 × g for 1 h at 4°C. The collected pellets were then dissolved in 1 M NaOH containing 10% DMSO at 80°C for 1 h. The absorbance of the heated solution was analyzed at 405 nm using a microplate reader.

### 2.5. Quantitative real-time polymerase chain reaction (qRT-PCR)

Culture medium containing α-MSH (200 nM) was added to B16F10 cells (1×10^5^ cells/well in a 6-well plate) for 1 h prior to treatment with AYC-P-E. After 24 h of AYC-P-E treatment, the cells were harvested, washed twice with cold PBS, and total RNA isolation and qRT-PCR were performed as previously described 22. Briefly, total RNA was extracted using the easy-spin Total RNA Extraction Kit (iNtRON Biotechnology, Seongnam, South Korea). Only RNA samples with a purity of 260/280 nm ratio 1.8 or higher were used in the experiment using Nanodrop (Maestrogen, Ramsey, MN, USA). cDNA was synthesized using 2 μg RNA and a high-capacity cDNA synthesis kit (Bioneer, Daejon, South Korea). Next, to measure the mRNA expression levels, the SYBR Green Mastermix (Applied Biosystems, Roche, USA) and the relevant primers were used to perform qRT-PCR using the Applied Biosystems 7500 Real-Time PCR instrument (Thermo Fisher Scientific). The following primers pairs were used for the qRT-PCR: Microphthalmia-associated transcription factor (MITF) forward, 5՛-CTGTACTCTGAGCAGCAGGTG-3՛ and reverse, 5՛- CCCGTCTCTGGAAACTTGATCG-3՛; Tyrosinase forward, 5՛-ATAACAGCTCCCACCAGTGC-3՛ and reverse, 5՛-CCCAGAAGCCAATGCACCTA-3՛; Tyrosinase related protein-1 (TRP-1) forward, 5՛-AGACGCTGCACTGCTGGTCAAGCCTGTAGCCCACGTCGTA-3՛ and reverse, 5՛-GCTGCAGGAGCCTTCTTTCT-3՛; Tyrosinase related protein-2 (TRP-2) forward, 5՛-GCTCCA AGTGGCTGTAGA-3՛ and reverse, 5՛-AATGCAGTGGCTTGGAAATC-3՛; and β-actin forward, 5՛-GCACCACACCTTCTACAATG-3՛. The thermal cycling protocol consisted of a hot start (40 cycles of 95°C for 5 min, 95°C for 20 sec, 60°C for 20 sec, and 72°C for 30 sec), and a melting curve (at 95°C for 1 min, 55°C for 1 min, and 30°C for 1 min). The relative expression of each gene was determined by normalizing its expression levels to those of β-actin.

### 2.6. Immunoblotting analysis

Culture medium containing α-MSH (200 nM) was added to B16F10 cells (1×10^5^ cells/well in a 6-well plate) for 1 h prior to treatment with AYC-P-E. After 24 h of AYC-P-E treatment, the cells were harvested, and washed twice with cold PBS. The washed cells were lysed using RIPA buffer, and proteins (20 µg) were subjected to 10% SDS-PAGE, and western blotting was performed as previously described 23. Cyclic AMP response element-binding protein (CREB), phosphorylated (p)-CREB (Ser^133^), tyrosinase, microphthalamia-associated transcription factor (MITF), tyrosinase related protein-1 (TRP-1), TRP-2, mitogen-activated protein kinase 1/2 (MEK1/2), p-MEK1/2 (Ser^218^/Ser^222^), extracellular signal-regulated kinase 1/2 (ERK1/2), p-ERK1/2 (Thr^202^/Tyr^204^), protein kinase B (PKB, also known as AKT), p-AKT (Ser^473^), and β-actin antibodies used were purchased from Santa Cruz Biotechnology (Dallas, TX, USA).

### 2.7. Clinical research

Clinical research was conducted by the Korea Dermatology Research Institute (KDRI; Bundang, South Korea) for eight weeks. Patients with skin hyperpigmentation on the face (22 female subjects aged 37 to 55 years with an average age of 49 years with phototype III or IV skin according to the Fitzpatrick phototype scale [Fitzpatrick, 1975]) were included in the study. The exclusion criteria were as follows: pregnant or lactating women and women who may be pregnant, patients with a history of photoallergies or photosensitization, those who had been using steroid-containing skin external preparations for more than 1 month, those who had participated in a similar study within 6 months of the start of the current study, those with sensitive and irritable skin, those with skin abnormalities, such as spots, acne, erythema, and expansion of capillaries, those who used the same or similar cosmetics or drugs on the same skin area within three months of the beginning of the study, and those who consumed medicine or food claimed to have skin-whitening effects. The clinical research was approved (KDRI-IRB-20275) by the Institutional Review Board (IRB) of the Korea Dermatology Research Institute, and this study was conducted at Seongnam, South Korea, in July to September 2020 in accordance with the WHO guidelines for good clinical practice (GCP) for trials on pharmaceutical products. All protocols were initiated after receiving written consent in compliance with the principles of the Helsinki Declaration.

The test samples were prepared in the form of a cream. In particular, the test sample contained 68.6% AYC-P-E, and the control group sample did not contain AYC-P-E. The samples were provided in the same container to ensure the double-blind design of the study. The test and control samples were randomly assigned to the left and right sides of the face of each subject, and the allocation was not disclosed to the subjects until the completion of the clinical research. The subjects applied each sample to the designated side of the face twice a day for 8 weeks. At each visit, all subjects washed the tested skin area and entered the constant temperature and humidity chamber (20-24°C, 40-60% RH) to rest for 30 min before participating in the test. The evaluation was conducted by measuring the melanin index (M-index) at the site of skin hyperpigmentation at each time point (2, 4, and 8 weeks) before and after using the sample. The melanin index was defined as the average value obtained by measuring the skin hyperpigmentation five times using a Mexameter® MX 18 (Courage+ Khazaka electronic, GmbH, Germany).

### 2.8. Statistical analysis

The* in vitro* data are expressed as the mean ± standard deviation (SD) of at least three independent experiments. The levels of significance for comparisons between samples were determined using one-way analysis of variance (ANOVA) followed by Tukey's multiple comparison test using the GraphPad statistical software (GraphPad Software, San Diego, CA). The clinical study data were analyzed using the Statistical Package for the Social Sciences (SPSS; IBM, USA) program. All data are expressed as the mean ± standard error of measurement (SEM). Normality and prior homogeneity for data analysis were verified using paired t-tests. The interdependence of the same subject, comparison between groups, and confirmation of changes before and after the indicated time points were analyzed using repeated-measures analysis of variance (RM ANOVA). A P-value of <0.05 was considered statistically significant.

## 3. Results

### 3.1. AYC-P-E decreases the intracelluar and extracelluar melanin contents in α-MSH-treated murine B16F10 melanoma cells

**Figure 1:**
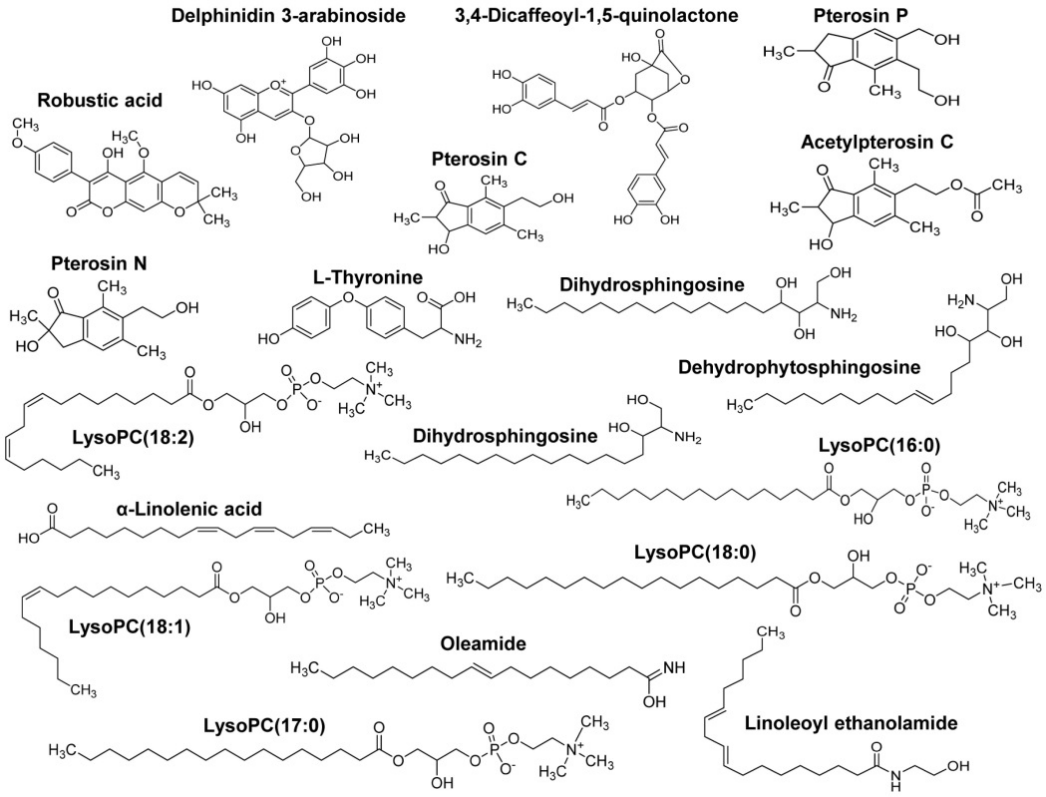
** Metabolite composition of AYC-P-E.** AYC-P-E includes 19 major metabolites, including one isoflavonoid (robustic acid), one flavonoid (delphinidin 3-arabinoside), four indanes (pterosin C, pterosin P, acetylpterosin C, and pterosin N), 1 cinnamic acid and derivative (3,4-Dicaffeoyl-1,5-quinolactone), one amino acid (L-Thyronine), three sphingolipids (dehydrophytoshingosine, dihydrosphingosine, and phytosphingosine), three fatty amides (α-Linolenic acid, linoleoyl ethanolamide, and oleamide), and five glycerophospholipids (LysoPC(18:2), LysoPC(16:0), LysoPC(18:1), LysoPC(17:0), and LysoPC(18:0)).

**Figure 2:**
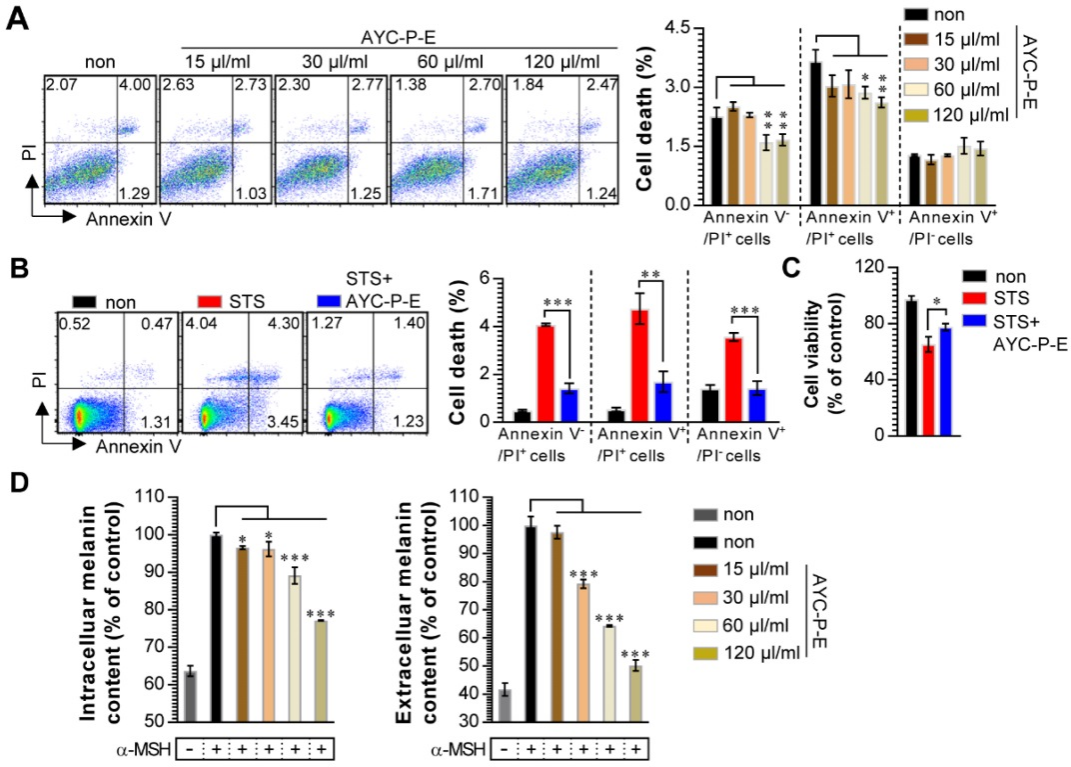
** Effect of AYC-P-E treatment on melanin synthesis in α-MSH-treated B16F10 cells.** (A and B) B16F10 cells were incubated with culture medium containing AYC-P-E at various concentrations (15, 30, 60, and 120 μl/ml) in the absence or presence of staurosporine (STS; 50 nM). After 72 h, cell viability and cell death (necrotic or apoptotic cell death) were measured using EZ-Cytox kit and Annexin V/PI staining, respectively. (A; left panel) The representative plot for Annexin V^+^/PI^+^, Annexin V^+^/PI^-^, or Annexin V^-^/PI^+^ B16F10 cells in AYC-P-E alone-treated condition. (A; right panel) The percentages of Annexin V^+^/PI^+^, Annexin V^+^/PI^-^, or Annexin V^-^/PI^+^ B16F10 cells in AYC-P-E alone-treated condition. (B; left panel) The representative plot for Annexin V^+^/PI^+^, Annexin V^+^/PI^-^, or Annexin V^-^/PI^+^ B16F10 cells in co-treatment condition with AYC-P-E (120 μl/ml) and STS (50 nM). (B; right panel) The percentages of Annexin V^+^/PI^+^, Annexin V^+^/PI^-^, or Annexin V^-^/PI^+^ B16F10 cells. (C) Cell viability in co-treatment condition with AYC-P-E (120 μl/ml) and STS (50 nM) was measured using EZ-Cytox kit. (D) The intracellular and extracellular melanin contents of B16F10 cells incubated with culture medium (phenol red-free) containing α-MSH (200 nM) for 1 h and treated with the indicated concentrations of AYC-P-E for 72 h. All bar graphs show the means ± standard deviation (SD) of three samples. One representative plot out of three independent experiments is shown; ^*^P<0.05, ^**^P<0.01, or ^***^P<0.001.

Our group recently showed the effectiveness of AYC-P-E in improving various phenomena, such as skin damage, inflammatory reaction, decreased type I procollagen, and increased elastase, accompanied by photoaging in UVB-irradiated keratinocytes 22. The present study aimed to evaluate the anti-melanogenic effects of the 19 metabolites present in AYC-P-E (Fig. 1) and the potential use of AYC-P-E in cosmeceutics as an anti-hyperpigmentation compound. Prior to conducting the clinical study of AYC-P-E in the treatment of hyperpigmentation, we first focused on determining the cellular toxicity of AYC-P-E in murine B16F10 melanoma cells. A low toxicity may offer new insight into the design and development of cosmeceutical ingredients. AYC-P-E cytotoxicity was analyzed by performing Annexin V and PI staining (Fig. 2A). We found that AYC-P-E is not cytotoxic as there were no remarkable differences in the percentage of dead cells (Annexin V^+^/PI^+^; late apoptotic cells, Annexin V^+^/PI^-^; early apoptotic cells, or Annexin V^-^/PI^+^ cells; necrotic cells) in B16F10 cultures exposed to AYC-P-E (15, 30, 60, and 120 μl/ml) for prolonged periods of time (72 h). We also confirmed the cytoprotective effect of AYC-P-E on the apoptosis inducer STS-treated melanoma cells (Fig. 2B and C). Interestingly, the B16F10 melanoma cells that were co-treated with AYC-P-E (120 μl/ml) and STS (50 nM) showed significantly reduced percentage of dead cells (late apoptotic cells, early apoptotic cells, and necrotic cells) compared with the STS alone-treated cells (Fig. 2B). Furthermore, STS-treated B16F10 melanoma cells significantly increased cell viability when treated with AYC-P-E (Fig. 2C), indicating that it does have a recovery effect against cellular damages of melanoma cells. Next, to investigate the effect of AYC-P-E on the regulation of melanogenesis in α-MSH*-*treated murine B16F10 melanoma cells, B16F10 cells were pretreated with α-MSH (200 nM) for 1 h prior to AYC-P-E treatment (15, 30, 60, and 120 μl/ml, for 72 h). Subsequently, we measured the intracellular and extracellular melanin contents. As shown in Fig. 2D, AYC-P-E decreased the intracellular and extracellular melanin contents induced by α-MSH stimulation in B16F10 cells in a dose-dependent manner. These results indicate that AYC-P-E can suppress the synthesis of melanin in α-MSH-stimulated B16F10 cells.

### 3.2. AYC-P-E downregulates the mRNA and protein expression of various factors related to melanogenesis

**Figure 3:**
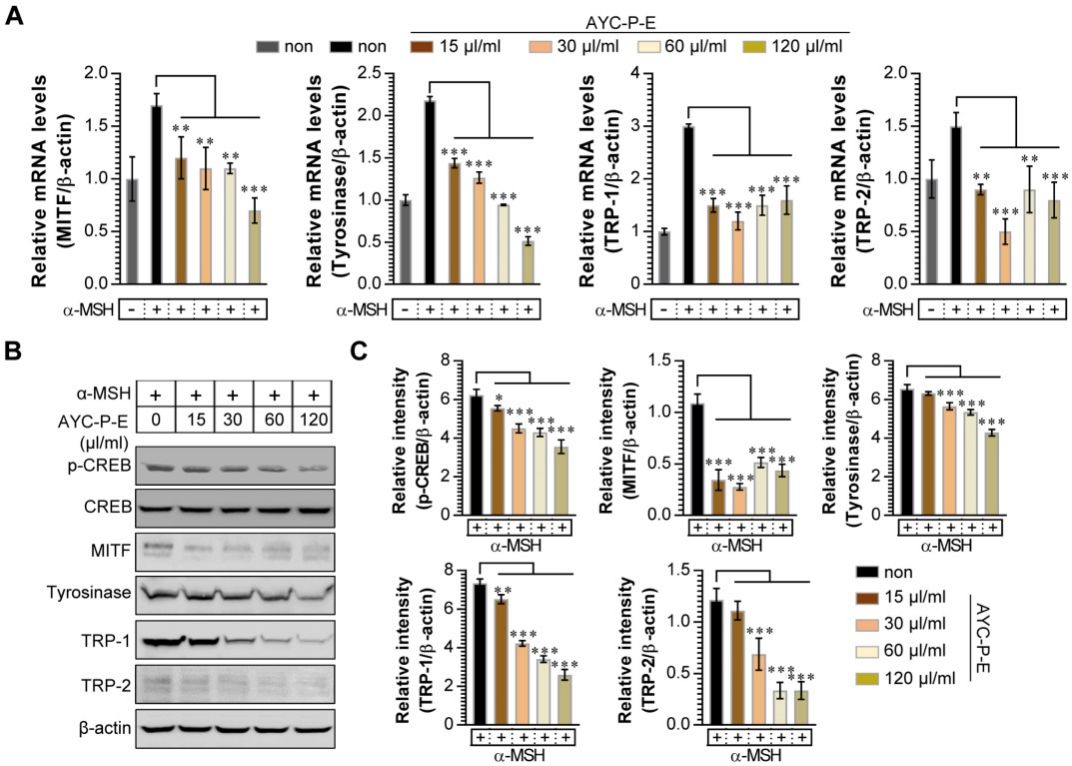
** Effect of AYC-P-E treatment on melanogenic protein and mRNA expression in α-MSH-stimulated B16F10 cells.** (A and B) B16F10 cells were incubated with culture medium containing α-MSH (200 nM) for 1 h and treated with the indicated concentrations of AYC-P-E for 24 h. (A) The mRNA expression of MITF, tyrosinase, TRP-1, and TRP-2 was analyzed using real-time PCR. (B) Immunoblot analyses were performed using Abs specific to p-CREB (Ser^133^), MITF, tyrosinase, TRP-1, and TRP-2. CREB and β-actin was used as the protein loading control. (C) The relative intensity of each protein level is expressed as the mean in the bar graphs. All bar graphs show the means ± standard deviation (SD) of three samples. One representative plot out of three independent experiments is shown; ^**^P<0.01, or ^***^P<0.001.

Melanogenesis is mediated by the activation of various signal transduction pathways, such as CREB, tyrosinase, MITF, TRP-1, and TRP-2 24. Here, CREB phosphorylation can lead to the activation of MITF expression, which is a transcription factor that plays a critical role in melanocyte development and differentiation. As a result, MITF binds to the promoter regions of the melanin production gene, leading thereby to stimulation (activation of tyrosinase, TRP-1, and TRP-2) of melanogenesis 25. Therefore, we evaluated whether AYC-P-E treatment inhibits the expression levels of p-CREB, MITF, TRP-1, and TRP-2 induced by α-MSH stimulation in B16F10 cells. As expected, treatment of α-MSH-stimulated B16F10 cells with AYC-P-E significantly inhibited the mRNA (Fig. 3A) and protein (Fig. 3B and C) expression levels of p-CREB, MITF, TRP-1, and TRP-2 compared to those of B16F10 cells treated with α-MSH alone. These results strongly suggest that AYC-P-E can exert anti-melanogenic effects by inhibiting the α-MSH-activated CREB, MITF, TRP-1, and TRP-2 signals in α-MSH-treated B16F10 melanoma cells.

### 3.3. AYC-P-E decreases melanogenesis through activation of ERK and AKT signaling

**Figure 4:**
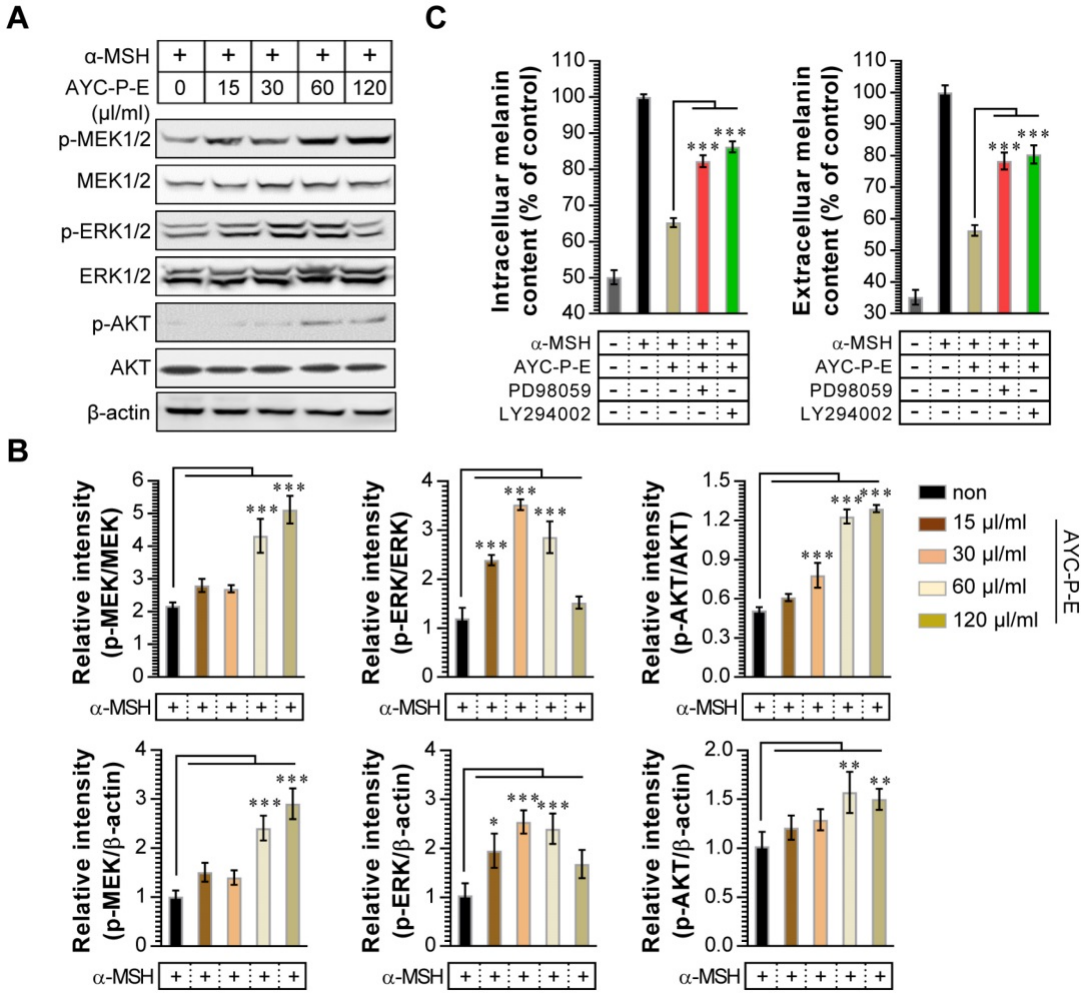
** Effect of AYC-P-E treatment on MEK/ERK and AKT signals in α-MSH-stimulated B16F10 cells.** (A) B16F10 cells were incubated in medium containing α-MSH (200 nM) for 1 h and treated with the indicated concentrations of AYC-P-E for 24 h. Immunoblot analyses were performed using Abs specific to p-MEK1/2 (Ser^217^/Ser^221^), p-ERK1/2 (Thr^202^/Tyr^204^), and p-AKT (Ser^473^). MEK1/2, ERK1/2, AKT, and β-actin antibodies were used as the protein loading control. (B; top panel) The relative intensity of each protein level compared with total loading controls (MEK1/2, ERK1/2, and AKT) is expressed as the mean in the bar graphs. (B; bottom panel) The relative intensity of each protein level compared with β-actin is expressed as the mean in the bar graphs. (C) B16F10 cells were incubated in medium containing α-MSH (200 nM) for 1 h, and treated with AYC-P-E (120 μl/ml) in the absence and presence of ERK (PD98059; 10 μM) and AKT (LY294002; 10 μM) inhibitors for 72 h. The intracellular and extracellular melanin contents were measured in the supernatant and pellets. All bar graphs show the means ± standard deviation (SD) of three samples. One representative plot out of three independent experiments is shown; ^*^P<0.05, ^**^P<0.01, or ^***^P<0.001.

Activation of MEK/ERK and AKT signaling phosphorylates MITF, resulting in melanogenesis inhibition. For example, p-MEK-mediated ERK activation (an increase of p-ERK expression) phosphorylates MITF at serine 73, which is followed by MITF ubiquitination and proteasome-mediated degradation 26. In addition, phosphorylation of AKT can decreases MITF expression via the inhibition of glycogen synthase kinase 3 beta, which downregulates melanogenesis 27. To examine whether these signals are involved in the anti-melanogenic effects following treatment with AYC-P-E, the activated (phosphorylated) MEK, ERK, and AKT signals were anlayzed using western blot analysis (Fig. 4A and B). We found that α-MSH-stimulated B16F10 cells exhibited significantly enhanced MEK, ERK, and AKT phosphorylation when treated with AYC-P-E compared to that of B16F10 cells treated with α-MSH alone. Next, to confirm the involvement of MEK/ERK and AKT signaling in the AYC-P-E-induced anti-melanogenic effect, we treated α-MSH-stimulated B16F10 cells with specific pharmacological inhibitors (PD98059 for MEK/ERK, LY294002 for AKT) and AYC-P-E and evaluated the intracelluar melanin contents following AYC-P-E treatment (Fig. 4C). These pharmacological inhibitors significantly abrogated the AYC-P-E-induced anti-melanogenic effect (decreased intracelluar melanin content) in α-MSH-stimulated B16F10 cells. Taken together, AYC-P-E was found to activate MEK/ERK and AKT signaling, which led to the suppression of melanin production.

### 3.4. Effect of AYC-P-E on skin pigmentation in a clinical setting

**Figure 5:**
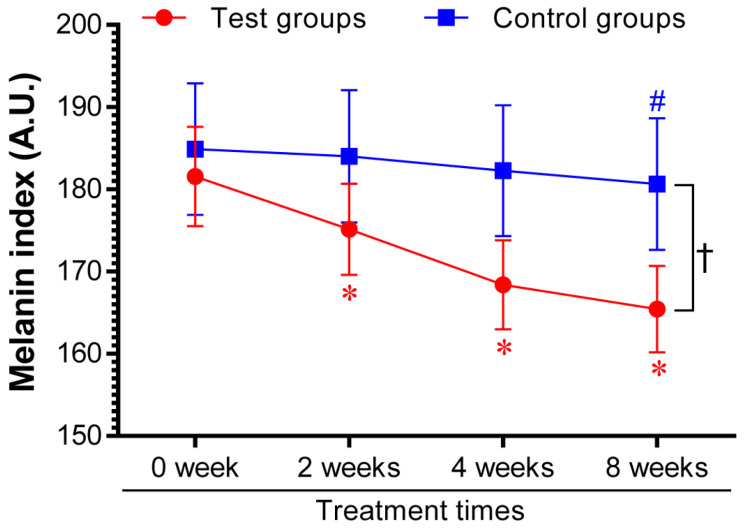
** Changes in melanin index following product application for eight consecutive weeks.** Twenty-two female patients with skin hyperpigmentation aged 37 to 55 participated in the study. The melanin index was analyzed at 2, 4, and 8 weeks after applying the test cream (Test groups) containing 68.6% AYC-P-E, and the control cream (Control groups) not containing AYC-P-E. Data from one of two independent experiments are shown. Data represent the mean ± standard error of measurement (SEM). ^*^P<0.05 compared to the melanin index before application (0 week) of the test cream containing AYC-P-E; ^#^P<0.05 compared to the melanin index before application (0 week) of the control cream not containing AYC-P-E; ^†^P<0.05 comparison of the melanin index of the test and control groups at 2, 4, and 8 weeks.

Based on the above* in vitro* results, the effectiveness of AYC-P-E in improving hyperpigmentation was examined in 22 subjects with hyperpigmentation in the facial area for 8 weeks after treatment with the test sample containing AYC-P-E and the control sample not containing AYC-P-E, and the change in the melanin index was analyzed. We found that the melanin index of the test group was significantly lower (P<0.05) than that of the control group 2 weeks after the initiation of AYC-P-E treatment and until the completion of the clinical study (8 weeks). The test group, treated with the test sample containing AYC-P-E, demonstrated 3.33, 7.06, and 8.68% hyperpigmentation improvement after 2, 4, and 8 weeks, respectively, whereas, the control group, treated with the control sample that did not contain AYCE, demonstrated 0.48, 1.41, and 2.5% hyperpigmentation improvement after 2, 4, and 8 weeks, respectively (Fig. 5). These results suggested that AYC-P-E could be used to improve skin pigmentation.

## 4. Discussion

This study aimed to assess the effects of AYC-P-E extracted from an *Aster yomena* callus culture system on melanogenesis *in vitro* and in clinical settings. We observed that AYC-P-E reduced melanin synthesis via the activation of the ERK and AKT signaling pathways in α-MSH-stimulated B16F10 melanoma cells. In a clinical study in 22 patients with hyperpigmentation, AYC-P-E also significantly improved the skin melanin level.

Melanin biosynthesis is promoted as a mechanism against skin damage caused by exposure to UV rays 28. Although melanin plays a positive role in protecting the skin, excessive melanin synthesis due to severe stimuli, such as UV rays may lead to pigmentation, such as freckles, and skin spots, and the toxicity of melanin precursors may lead to cell death and skin cancer 28, 29. In fact, keratinocyte-derived mediators, such as α-MSH, basic fibroblastic growth factor, stem cell factor, endothelin-1, produced by UV stimulation can result in the activation of melanocytes, consequently promoting melanogenesis 30, 31. Interestingly, we recently observed that AYC-P-E exerts anti-photoaging functions in UVB-irradiated human keratinocytes by improving cell viability, inhibiting elastase, forming Type I procollagen, and inhibiting TNF-α. AYC-P-E is consists of 19 main metabolites including one isoflavonoid, one flavonoid, and three sphingolipids 22. Among these, flavonoid compounds, such as isoflavonoids and flavonoids, have strong anti-oxidative, anti-carcinogenic, as well as anti-inflammatory effects 32, 33. In particular, robustic acid is capable of inducing anti-tumor activity, while among sphingolipids, phytosphingosine plays an important role in the regeneration of damaged skin 23,24. Although the anti-photoaging and whitening effects of each metabolite contained in AYC-P-E have not yet been identified, based on the above findings, the AYC-P-E-induced inhibition of melanin synthesis may be mediated by the presence of such metabolites.

Skin pigmentation is induced by melanogenesis, a complex process involving tyrosinases and other tyrosinase-related proteins (TRPs) in melanocytes 34. Therefore, the mechanism of action of skin whitening agents is associated with reduced tyrosinase activity, which subsequently leads to the inhibition of melanogenesis 35. Importantly, the expression of tyrosinase, TRP-1, and TRP-2 is induced by the activation of the cAMP/PKA/CREB signaling pathway which further increases MITF expression 36. MITF is a master gene essential to the development and survival of melanocytes 37. In this study, the anti-melanogenic activity of AYC-P-E induced the phosphorylation of CREB, which was accompanied by the α-MSH-induced inhibition of MITF in melanocytes. As a result, the mRNA and protein levels of tyrosinase, TRP-1 and TRP-2 decreased. Moreover, AYC-P-E treatment of α-MSH-stimulated B16F10 cells led to MEK/ERK and AKT phosphorylation. The MEK/ERK and AKT signaling pathways are closely related to MITF transactivation and stability 38. For example, the activation of the MERK/ERK and AKT signaling pathways can promote the degradation of MITF in melanocytes that induce melanogenesis, thus resulting in anti-melanogenic activity 39, 40. In fact, following the treatment of α-MSH-stimulated B16F10 cells with AYC-P-E and specific ERK and AKT inhibitors, the inhibitory effect of AYC-P-E on melanin synthesis was impaired. Therefore, the clinical effects of improved melanin levels in subjects with hyperpigmentation seem to be mediated by MITF degradation via the activation of ERK and AKT, accompanied by the subsequent inhibition of tyrosinase and other tyrosinase-related proteins.

In this study, inhibition of melanin synthesis by AYC-P-E was mediated by ERK and AKT activation, which resulted in improved melanin levels in patients with hyperpigmentation. This suggested the potential role of AYC-P-E as a functional cosmetic ingredient in skin whitening procedures or formulations. However, the whitening effects of each metabolite in AYC-P-E were not assessed. AYC-P-E contains a number of metabolites whose functionality has not been revealed. Therefore, further studies are required to identify the key metabolites of AYC-P-E possessing this whitening activity.

## 5. Conclusions

In conclusion, AYC-P-E extracted using a plant tissue culture technique induced MITF degradation followed by downregulation of melanin-inducing factors (tyrosinase, TRP-1 and TRP-2) in melanocytes that promote melanogenesis. Importantly, AYC-P-E inhibited melanogenesis by activating the ERK and AKT signaling pathways. In conclusion, our previous findings as well as the results of the present study on the anti-photoaging effects of AYC-P-E indicate that AYC-P-E may be used as an ingredient in functional cosmetic products for the treatment of various symptoms (including wrinkles, damaged skin barrier, hyperpigmentation, etc.) caused by photoaging.

## Abbreviations

AYC-P-E: *Aster yomena* callus pellet extract
α-MSH: α-melanocyte stimulating hormone
CREB: cyclic AMP response element-binding protein
MITF: microphthalmia-associated transcription factor
TRP-1: tyrosinase related protein-1
TRP-2: tyrosinase related protein-2
MEK: mitogen-activated protein kinase
ERK: extracellular-signal-regulated kinase
AKT: protein kinase B
qRT-PCR: quantitative real-time polymerase chain reaction
PBS: Phosphate-buffered saline
ELISA: Enzyme-linked immunosorbent assay
SD: standard deviation
SEM: standard error of measurement
